# A new species of *Calogalesus* Kieffer from China (Hymenoptera, Diapriidae) with a key to World species

**DOI:** 10.3897/zookeys.626.9771

**Published:** 2016-10-20

**Authors:** Jun Feng, David Notton, Zai-fu Xu

**Affiliations:** 1 Department of Entomology, South China Agricultural University, Guangzhou 510640, P. R. China; 2 Department of Life Sciences, Insects Division, Darwin Centre - room 315, The Natural History Museum, Cromwell Road, London, SW7 5BD, United Kingdom

**Keywords:** Hymenoptera, Diapriinae, Calogalesus, new species, Oriental region, China

## Abstract

A new species of *Calogalesus* Kieffer, 1912, *Calogalesus sinicus* **sp. n.**, is described and illustrated, collected from a Chinese prickly ash (*Zanthoxylum bungeanum* Maxim.) orchard in Yunnan province of China. This is the third described species of the genus in the World. The new species can be distinguished from the other two described *Calogalesus* species by the head profile, proportions of the antennal segments, tridentate mandible, and mandible length. A key to World species of the genus is provided.

## Introduction

*Calogalesus* Kieffer, 1912 is a small genus in Diapriinae (Hymenoptera: Diapriidae), comprising two previously described species: *Calogalesus parvulus* Kieffer, 1912 from the Seychelles and *Calogalesus malabaricus* Rajmohana & Narendran, 2006 from India (Kieffer 1912a; Rajmohana and Narendran 2006). Its ecology and biology are unknown.

In recent years, during the survey of the Chinese fauna of Diapriidae funded by the National Natural Science Foundation of China, fifty-four specimens (of both sexes) belonging to *Calogalesus* were collected in Yunnan; this material is described here as a new species.

## Materials and methods

All specimens were collected using yellow pan traps placed in a Chinese prickly ash (*Zanthoxylum bungeanum* Maxim.) orchard in Zhaotong, Yunnan from 10.VIII.2012 to 26.X.2012.

Specimens were examined and described under a Zeiss Stemi 2000-CS stereomicroscope. All photos were taken with a digital camera (Cool SNAP) attached to the Zeiss Stemi 2000-CS stereomicroscope and processed by using Image-Pro Plus software.

Morphological terminology mainly follows Masner and García Rodríguez (2002). The following abbreviations are used: A1, A2, … = the first, second, .... antennal segments, respectively; OOL = the shortest distance between posterior ocellus and compound eye; POL = the shortest distance between both posterior ocelli; T2 = the second (largest) tergite.

Measurements reported are relative, and refer to ratios, except for body length (head to abdominal tip, excluding antennae and ovipositor, when the body is fully extended) and fore wing length.

The holotype, 13 female and 34 male paratypes of the new species are deposited in the Hymenoptera Collection of South China Agricultural University, Guangzhou, Guangdong province, China (SCAU); three female and three male paratypes are deposited in The Natural History Museum, London, UK (NHMUK). The holotype ♀ of *Calogalesus parvulus* Kieffer, 1912 from the Seychelles (NHMUK010264968) and one male of *Calogalesus malabaricus* Rajmohana & Narendran, 2006 from India (Karnataka, Mudigere, 26.x–4.xi.1979, J. S. Noyes leg. (NHMUK010264967)) were compared with the new species.

## Results

### Genus Calogalesus

Taxon classificationAnimaliaHymenopteraDiapriidae

Kieffer, 1912

Calogalesus Kieffer, 1912b: 6, 43. Type species: Calogalesus parvulus Kieffer, 1912, by monotypy; Kieffer 1912a: 73; Kieffer 1916: 10, 235; Muesebeck and Walkley 1956: 338; Johnson 1992: 145; Masner and García Rodríguez 2002: 116; Rajmohana 2006; 36.
Calicuta Rajmohana & Narendran, 2000a: 22–23, unavailable name; Rajmohana and Narendran 2000b: 193; Rajmohana & Narendran, in Rajmohana 2004: 519, 521; Rajmohana 2006: 36.

#### Diagnosis.

Body mainly blackish-brown, brown or orange, smooth and shiny. Head with antennal shelf strongly projecting, laterally sharply angled and medially divided; frons with a curved carina on each side extending backwards, forming a ledge above upper eye orbit. Mandible bidentate or tridentate, together beak-like, projecting backwards. Antenna 12-segmented in female, 14-segmented in male; with A1 the longest segment. Female antenna without a clearly defined clava, but flagellar segments more or less thickened apically. Notauli distinct, not reaching transscutal articulation. Scutellum with two large anterior foveae. Fore wing with well-developed marginal cilia and two elongate hairless zones. Petiole strongly curved in lateral view.

#### Biology.

This genus shows a nasiform head (elongated with frontal projections) and opisthognathous (backwards directed) beak-like mandibles, which may be associated with digging for hosts and/or bursting from host remains (Nielsen and Buffington 2011).

#### Distribution.

The genus is known from the following biogeographic regions: Afrotropical (Masner and García Rodríguez 2002); Australian (Masner and García Rodríguez 2002); Malagasy (Kieffer 1912a, 1912b, 1916; Masner 1965; Gerlach 2013; Notton 2014; Madl 2015); Neotropical (Masner and García Rodríguez 2002; Arias-Penna 2003); Oriental (Rajmohana 2004, 2006; Rajmohana and Narendran 2000a, 2000b; Rajmohana and Bijoy 2012; Rajmohana et al. 2013).

#### Remarks.

The genus *Calicuta* was described by Rajmohana and Narendran (2000a) without type species designation, without included species and was not explicitly indicated as new. It is therefore an unavailable name. A formal publication of the name was intended but was abandoned following the realization that it was the same genus as *Calogalesus* Kieffer (Rajmohana 2006; Rajmohana pers. comm. with Notton). The name *Calicuta* was not made available by any of the subsequent publications of Rajmohana and Narendran cited here.

**Key to World species of *Calogalesus***

**Table:** 

1	Females: 12 antennal segments; apex of metasoma pointed; ovipositor sheaths visible; apical flagellar segments more or less thickened	**2**
–	Males: 14 antennal segments; apex of metasoma blunt, often sunk inside T2 and not visible; no ovipositor sheaths; apical flagellar segments usually elongate, at least not thicker than basal flagellar segments	**4**
2	Antenna longer than head and mesosoma (1.1:1.0), A4–A7 1.4 times as long as wide, A8–A10 1.17 times as long as wide; head profile less flattened (head length to height=1.0:1.0), subtriangular in lateral view; mandible tridentate, 0.5 times as long as eye height; POL:OOL=0.4:1.0	***Calogalesus sinicus* sp. n.**
–	Antenna shorter than head and mesosoma (0.85–0.9:1.0), A4–A7 as long as wide, A8–A10 as long as wide or shorter than wide; head profile more flattened (head length to height=1.2–1.3:1.0), subrectangular in lateral view; mandible bidentate, 0.6–0.8 times as long as eye height; POL:OOL=1.2–1.3:1.0	**3**
3	A2 as long as A3; A8–A10 as long as wide; mandible 0.8 times as long as eye height	***Calogalesus malabaricus* Rajmohana & Narendran**
–	A2 1.5 times as long as A3; A8–A10 distinctly shorter than wide; mandible 0.6 times as long as eye height (Fig. 10)	***Calogalesus parvulus* Kieffer**
4	Head profile more flattened (head length to height=1.3:1.0), subrectangular in lateral view; mandible nearly as long as eye height (0.8:1.0); A4 0.9 times as long as A3	***Calogalesus malabaricus* Rajmohana & Narendran**
–	Head profile less flattened (head length to height=1.0:1.0), subtriangular in lateral view; mandible distinctly shorter than eye height (1.0:2.0); A4 0.8 times as long as A3	***Calogalesus sinicus* sp. n.**

### Calogalesus sinicus

Taxon classificationAnimaliaHymenopteraDiapriidae

Feng, Notton & Xu sp. n.

http://zoobank.org/0A2A49D5-D787-4006-A407-ED4F252B126E

Figs 1–2 , 3–4 , 5 , 6–7 , 8–9

#### Material examined.

Holotype, ♀: CHINA: Yunnan, Zhaotong, Huanghua town (N27°59', E103°33'), 10.VIII.2012, Shi-wen Yang leg. Paratypes: 16 ♀♀, Yunnan, Zhaotong, Huanghua town (N27°59', E103°33'), 10.VIII–26.X.2012, Shi-wen Yang leg.; 37 ♂♂: Yunnan, Zhaotong, Huanghua town (N27°59', E103°33'), 10.VIII–26.X.2012, Shi-wen Yang leg.

#### Description.

**Figures 1–2.**
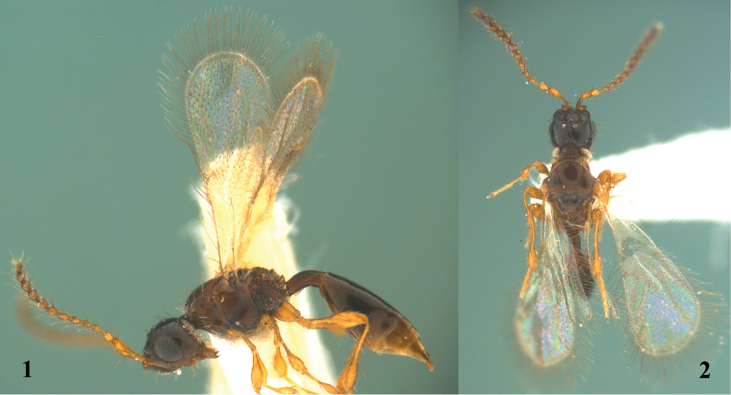
*Calogalesus sinicus* sp. n., ♀, holotype, habitus. **1** Lateral **2** dorsal.

**Figures 3–4.**
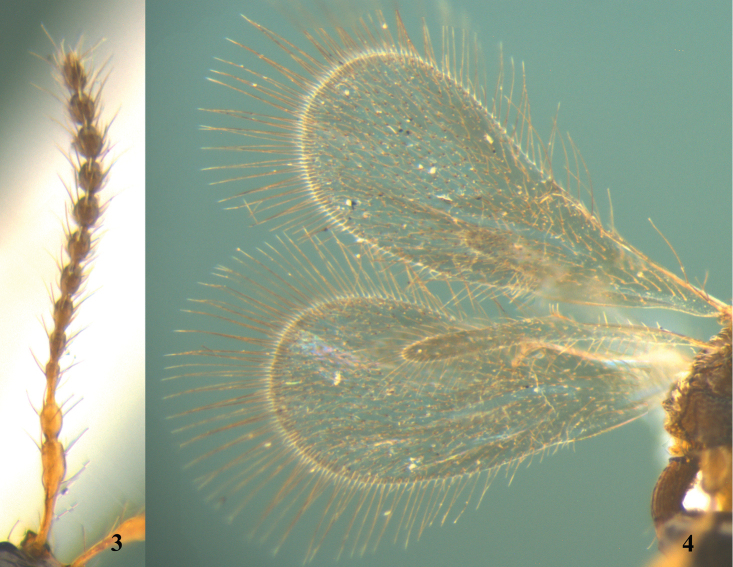
*Calogalesus sinicus* sp. n., ♀, holotype. **3** Antenna **4** wings.

*Female* (Figs 1–4). Holotype. Body length 1.2 mm. Fore wing length 1.0 mm.

*Colour*. Head blackish-brown, mandibles brown. Antennae yellowish-brown. Mesosoma brown. Legs and tegulae yellowish-brown. Wings hyaline, with veins yellowish-brown. Metasoma brown, with apex yellowish-brown.

*Head*. Head subcircular in dorsal view, subtriangular in lateral view; smooth and shiny, with sparse hairs. Mandible tridentate, strongly projecting, beak-like. Labrum subtriangular. Clypeus highly convex. Eyes oval, slightly bulging laterally, with sparse hairs. Malar sulcus distinct. Frons with two sharp points and longitudinal ledges above upper eye orbit. Antennal shelf strongly projecting. Antenna 12-segmented (Fig. 3), with hairs slightly longer than width of antennal segment. Relative proportion of length to width of antennal segments: A1 (24:6); A2 (7:5); A3 (10:4); A4 (7:4); A5 (7:5); A6 (7:5); A7 (7:5); A8 (7:6); A9 (7:6); A10 (7:6); A11 (7:6); A12 (8:5). Eye 1.5 times as long as wide, 2.5 times as long as malar space. Ocelli with POL:OOL=5:12. Occipital flange moderately developed and step-like. Genal ridge with tufts of hairs.

*Mesosoma*. Mesosoma as wide as head in dorsal view. Cervix distinct. Pronotum smooth and shiny. Pronotal shoulders almost rounded. Epomium indistinct. Mesoscutum smooth and shiny, with sparse hairs. Notauli deep and posteriorly convergent, incomplete, reaching 0.90 length of mesoscutum, not reaching transscutal articulation. Humeral sulcus distinct. Mesoscutellum subtriangular, smooth and shiny, with two large foveae on anterior half and a row of small pits on the posterior margin. Mesopleuron smooth and shiny, with a groove beneath tegula. Sternaulus indistinct. Metanotum and metapleura reticulate rugose. Propodeum reticulate rugose. Dorsal surface of propodeum with one median longitudinal keel. Plica distinctly projecting posteriorly. Posterior surface of propodeum carinate and emarginated, descending abruptly and steeply with two postero-lateral teeth in lateral view. Wings (Fig. 4) fully developed, with long marginal cilia. Fore wing with two elongate hairless zones basally; costal, subcostal, marginal and stigmal veins present, basal and postmarginal veins absent. Venation extending to half length of fore wing. Stigmal vein elongate, 0.6 times as long as marginal vein. Hind wing with submarginal vein complete. Femora and tibiae clavate. Fore tibia without an outwardly directed spine.

*Metasoma*. Petiole sparsely hairy, shiny, rugose with longitudinal striae, distinctly curved in lateral view; 2.6 times as long as wide in dorsal view; 2.3 times as long as high in lateral view. Gaster moderately compressed laterally. T2 enlarged, 2.6 times as long as wide in dorsal view, and covering 0.65 length of gaster in dorsal view. Anterior margin of T2 straight, without furrow or emargination. Metasomal tip conical.

*Variation*. Body length 1.1–1.4 mm (n=17). Fore wing length 1.0–1.3 mm (n=17).

**Figure 5.**
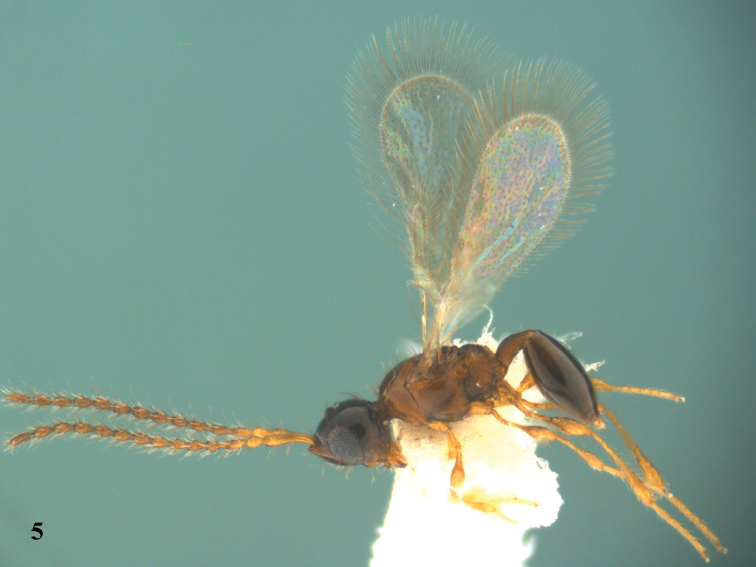
*Calogalesus sinicus* sp. n., ♂, paratype, habitus, lateral.

**Figures 6–7.**
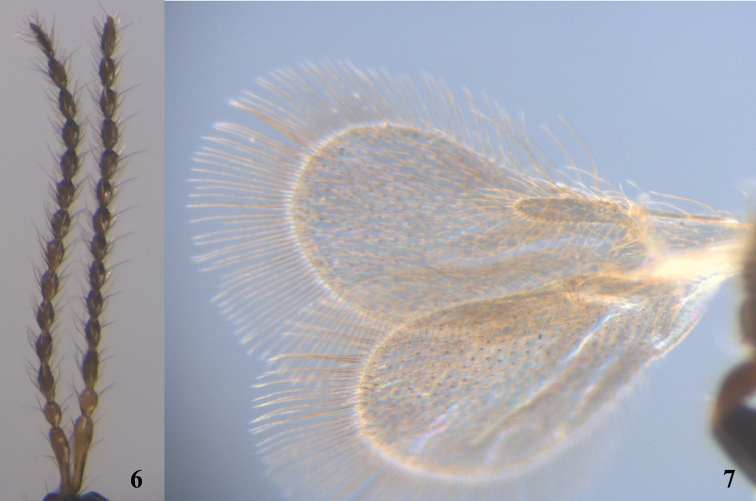
*Calogalesus sinicus* sp. n., ♂, paratype. **6** Antennae **7** wings.

*Male* (Figs 5–7). Body length 1.0–1.3 mm (n=37). Fore wing length 0.8–1.2 mm (n=37). Antenna 14-segmented (Fig. 6), with hairs slightly longer than width of antennal segment. Relative proportion of length to width of antennal segments as follows: A1 (23:4); A2 (7:4); A3 (10:3); A4 (8:3); A5 (7:4); A6 (7:4); A7 (7:4); A8 (7:4); A9 (7:4); A10 (7:4); A11 (7:4); A2 (7:4); A3 (7:4); A14 (8:4). A4 not modified. Metasomal tip blunt. Other characteristics as for females.

#### Biology.

**Figures 8–9.**
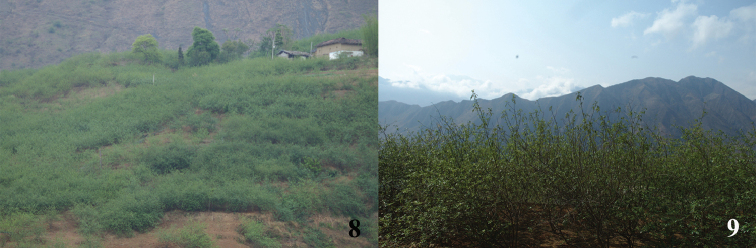
Habitat of *Calogalesus sinicus* sp. n. **8** Chinese prickly ash (*Zanthoxylum bungeanum* Maxim.) orchard in Huanghua town, Zhaotong city, Yunnan province **9** *Zanthoxylum bungeanum* Maxim. (Photos by Wei Dong)

Host unknown. This species was collected by placing 500 yellow pan traps in a Chinese prickly ash (*Zanthoxylum bungeanum* Maxim.) orchard (Figs 8, 9) from 10.VIII.2012 to 26.X.2012. Specimens were picked up every day.

#### Distribution.

Known from a single location in China (Yunnan).

#### Remarks.

**Figure 10.**
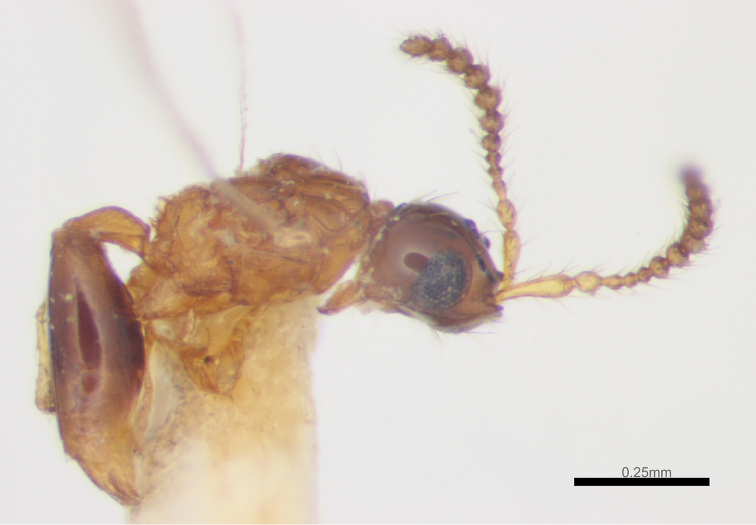
*Calogalesus parvulus* Kieffer, 1912, ♀, holotype, habitus, lateral. (Photo by Zi Hou, © The Trustees of the Natural History Museum, London)

This new species can be separated from the two described species, *Calogalesus parvulus* Kieffer, 1912 (Fig. 10), and *Calogalesus malabaricus* Rajmohana & Narendran, 2006, by the characters given in the key. In all previous descriptions of *Calogalesus* (Masner and García Rodríguez 2002; Rajmohana et al. 2013), state that the mandible are bidentate, however the new species has tridentate mandibles, so we have revised the generic diagnosis accordingly.

#### Etymology.

The new species is named after the country of the type locality, China.

## Supplementary Material

XML Treatment for Calogalesus

XML Treatment for Calogalesus sinicus
